# Combining Cisplatin with Different Radiation Qualities—Interpretation of Cytotoxic Effects In Vitro by Isobolographic Analysis

**DOI:** 10.3390/ph16121720

**Published:** 2023-12-12

**Authors:** Roswitha Runge, Falco Reissig, Nora Herzog, Liane Oehme, Claudia Brogsitter, Joerg Kotzerke

**Affiliations:** 1 Department of Nuclear Medicine, University Hospital Carl Gustav Carus, Technical University Dresden, Fetscherstr. 74, 01307 Dresden, Germany; nora.herzog@uniklinikum-dresden.de (N.H.); claudia.brogsitter@uniklinikum-dresden.de (C.B.); joerg.kotzerke@uniklinikum-dresden.de (J.K.); 2 Helmholtz-Zentrum Dresden-Rossendorf, Institute of Radiopharmaceutical Cancer Research, Bautzner Landstraße 400, 01328 Dresden, Germany; f.reissig@hzdr.de

**Keywords:** cisplatin, radionuclides, alpha-emitter, cancer therapy, combined treatment, isobolograms

## Abstract

Background: The combination of platinum-containing cytostatic drugs with different radiation qualities has been studied for years. Despite their massive side effects, these drugs still belong to the therapeutic portfolio in cancer treatment. To overcome the disadvantages of cisplatin, our study investigated the cytotoxic effects of combining radionuclides with cisplatin. Methods: FaDu cells were treated with cisplatin (concentration ≈ 2 µM) and additionally irradiated after two hours with the alpha-emitter ^223^Ra, the beta-emitter ^188^Re as well as external X-rays using dose ranges of 2–6 Gy. Cell survival was followed by colony formation assays and plotted against cisplatin concentration and radiation dose. The results were interpreted by isobolograms. Results: Isobolographic analyses revealed a supra-additive cytotoxic effect for the combination of cisplatin and ^223^Ra. A sub-additive effect was observed for the combination of cisplatin and ^188^Re, whereas a protective effect was found for the combination with X-rays. Conclusions: The combination of cisplatin and ^223^Ra may have the potential to create a successfully working therapy scheme for various therapy approaches, whereas the combination with ^188^Re as well as single-dose X-ray treatment did not lead to a detectable radiosensitizing effect. Thus, the combination with alpha-emitters might be advantageous and, therefore, should be followed in future studies when combined with cytostatic drugs.

## 1. Introduction

Cisplatin (cis-diammine-dichloro-platinum (II), CDDP) and its derivatives, carboplatin and oxaliplatin, have been widely used in the treatment of human cancers, such as bladder, head and neck, lung, ovarian, and testicular carcinomas [1]. Their mechanism of action is based on direct interactions of the platinum complexes within the DNA strands, resulting in the inhibition of cell repair mechanisms and thereby reducing tumor volume. In the past, the general use of platinum pharmaceuticals in cancer treatment focused on the rather unselective therapy of different tumor tissues [2,3]. Platinum drugs are used as a first-line treatment for solid tumors when radiation is not an option or as a second-line treatment in combination with other chemotherapeutic agents. The major limitations in the use of cisplatin and its derivatives are the renal side effects as well as the development of resistance in cancer cells [4].

In the past, extensive clinical studies have demonstrated the high efficacy of targeted radionuclide therapies such as ^177^Lu-PSMA-617 radioligand therapy for metastatic castration-resistant prostate cancer (VISION) [5]; ^177^Lu-DOTATATE for progressive midgut neuroendocrine tumors (NETTER-1) [6]; and radionuclide therapy with radium-223-dichloride, selectively targeting bone metastases from prostate cancer (ALSYMPCA) [7]. Subsequently, the successful proof of concept for targeted alpha therapy (TAT) has led to further research interest in this area [8,9]. In addition, the clinical applications of TAT are continuously growing, with different radionuclides, such as ^225^Ac, ^212^Pb, and ^227Th^, as promising candidates. Alpha-particles are characterized by high linear energy transfer (LET), leading predominantly to a direct radiation response and impaired DNA repair [10].

To overcome the normal tissue side effects, the focus has been directed on the optimization of commonly used approaches, namely cisplatin-based chemo-radiotherapy. In recent years, several radiosensitizing effects have been reported, providing promising data. In particular, increased DNA damage has been observed in tumor cells after the combination of platinum drugs and external irradiation [11,12,13,14]. Numerous clinical studies have been performed on this topic. Marcu et al. proposed techniques to increase the tumor control probability (TCP) of head and neck cancer while protecting normal tissue in a phase II study [15]. In addition, a higher rate of complete pathologic response was observed in patients treated with platinum-based chemo-radiotherapy compared with patients receiving chemotherapy alone [12].

Additionally, the preclinical evaluation of nanoparticle encapsulated cisplatin (BNC-LP-CDDP) as chemo-radiotherapy treatment revealed the elimination of the nephrotoxic properties in vitro and in vivo, and thus, the nanoparticles can improve the local deposition of higher doses in the target region [16]. Sisin et al. found increased efficacy of tumor control for the combination of cisplatin and bismuth oxide nanoparticles (BiONP) on MCF-7 cells under ^192^Ir-high dose rate brachytherapy [17].

It may be advantageous to combine cytostatics with different radiation qualities, such as beta and alpha radiation, as well as external X-rays. Due to the special physical properties of radionuclides, their low-dose rate of radiation is an important aspect when considering the high-dose rate of X-rays. Thus, the biological effects assessed from radionuclide exposure may, at least in part, underly different signaling pathways than those induced by X-rays.

To date, only a few studies have been performed using radionuclides that are commonly used in nuclear medicine [18,19,20,21]. The enhancement of radiobiological effects of radiometals, especially the underlying mechanism of Auger effects, has been reviewed by Kobayashi et al. and Nias et al. [22,23]. Another interesting aspect of platinum is the availability of radioactive platinum isotopes, which would even allow the use of a radiolabeled platinum complex. The DNA-destructive effect of radioactive platinum isotopes has also been reported [22,24,25]. 

Overall, the growing interest in the use of radiotherapy, especially the TAT approaches, indicates the need for further basic research in this area.

As a consequence, our investigations may contribute to discovering if the chemo-radiotherapeutic approach has the potential for successful application in cancer therapy.

In this particular study, human-derived head and neck cancer cells (FaDu) were incubated with cisplatin and additionally irradiated with ^233^Ra as alpha-emitter, ^188^Re as beta-emitter, or external X-rays. Cytotoxicity was measured by observing cell viability using colony formation assays. Results were interpreted by isobolographic analyses to distinguish between supra-additive, additive, or protective effects [26,27].

## 2. Results

### 2.1. Dose–Response Curves of Single Cytotoxin Incubations

**Figure 1:**
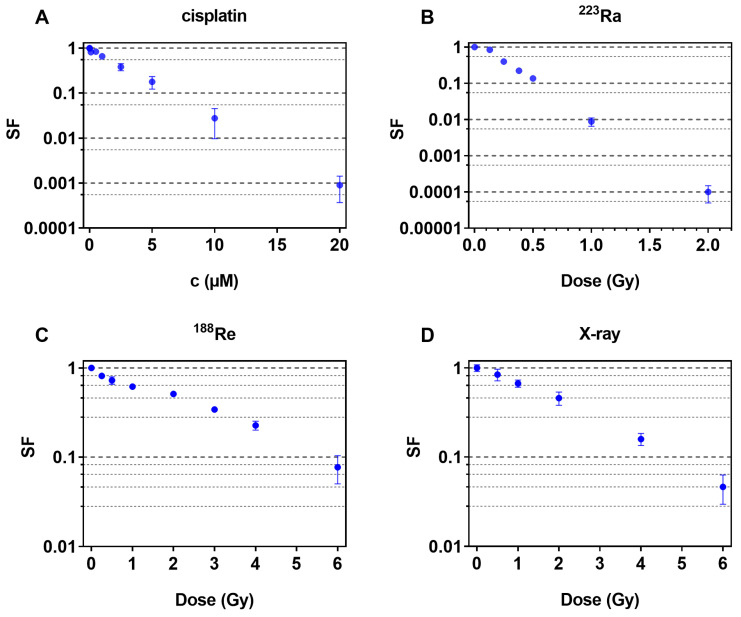
Cell survival fractions of FaDu cells after exposure to cisplatin (**A**), ^223^Ra (**B**), ^188^Re (**C**) or X-ray (**D**). Data are expressed as mean and standard deviation (±SD).

To evaluate the damaging potential of the cytostatic drug and all radiation qualities, single incubations were performed on FaDu cells, and dose–response relationships were measured via clonogenic cell survival. The dose–response curves are shown in Figure 1.

A clear dependence of the cell survival on concentration and radiation dose was observed for all different cytotoxins—cisplatin and radionuclides. In particular, the incubation of ^223^Ra seems to cause more damage compared to ^188^Re and X-rays at similar doses.

Additionally, the cisplatin concentrations that reduced the survival fractions to 0.37 or 0.50 (C_37_, C_50_) and the corresponding radiation doses D_37_ and D_50_ were calculated and are shown in Table 1.

Cisplatin, X-rays, and ^188^Re show similar D_37_ and D_50_ values, whereas about 10-fold-lower D_37_ and D_50_ values were observed for ^223^Ra.

### 2.2. Dose–Response Curves of Combined Treatments

To determine the nature of the interaction between cisplatin and radiation, isobolograms were constructed for two survival levels (SF = 0.37 or SF = 0.5). The interactions become supra-additive (radiosensitizing) when the effect of the combined therapy is greater than the sum of the responses of the respective single agents. Mode I represents the simple additivity of responses, while Mode II takes into account dose additivity as described in Section 4.

#### 2.2.1. Combination of Cisplatin and ^223^Ra

**Figure 2:**
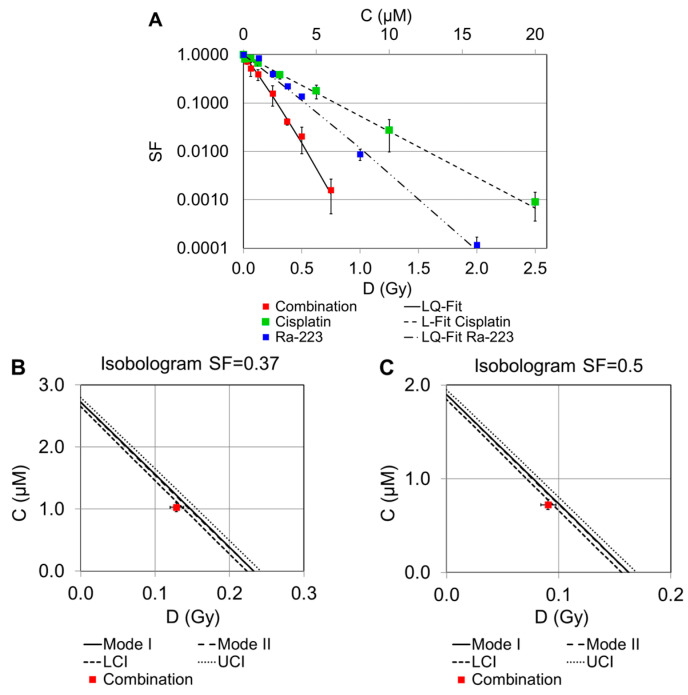
Survival curves of single and combined treatment experiments with cisplatin and ^223^Ra are displayed, and the measured values are shown as mean ± SD (**A**). Isobolograms are displayed for survival fractions 0.37 (**B**) and 0.50 (**C**). The solid line represents the line of additivity; the dashed lines are the calculated errors with respect to the lower (LCI) and upper (UCI) 95% confidence intervals.

Combination experiments of cisplatin and ^223^Ra at certain concentration ratios are displayed in Figure 2A. It can be seen that the combined treatment resulted in a significant reduction in the survival fractions. The measured values for ^223^Ra and the combination were fitted according to the linear–quadratic model (LQ-Fit). The theoretical isoeffective curves for the combination of both agents were calculated for Mode I and II (see Section 4). However, the resulting lines for these two agents are almost indistinguishable. Isobolograms for the interaction of both drugs are shown in Figure 2B,C. According to Figure 2A, the combination of cisplatin and ^223^Ra caused a small supra-additive effect. It can be assumed that a cisplatin-induced blockade of DNA replication and DNA repair enhances cell death, leading to an increase in irreparable damage to DNA, which in turn causes cell death [28,29]. It is well known that the efficiency of radiation therapy depends on the early induction of cell damage (apoptosis), and thus, the alpha-emitter ^223^Ra may enhance cell death more efficiently than ^188^Re or X-rays as radiation characterized by low-LET emitters [30].

#### 2.2.2. Combination of Cisplatin and ^188^Re

**Figure 3:**
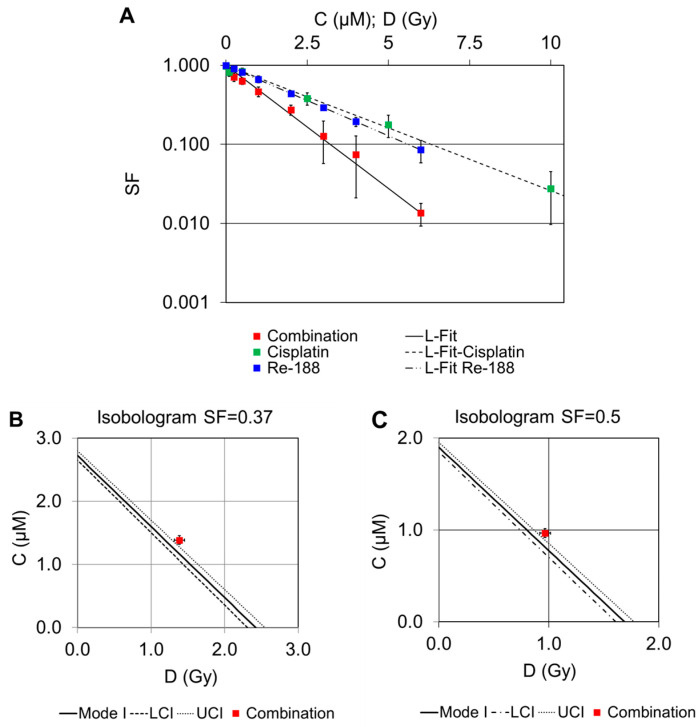
Survival curves of single and combined treatment experiments with cisplatin and ^188^Re are displayed, and the measured values are shown as mean ± SD (**A**). Isobolograms show survival fractions of 0.37 (**B**) and 0.50 (**C**). The solid line represents the line of additivity; the dashed lines are the calculated errors in terms of the lower (LCI) and upper (UCI) 95% confidence intervals.

Combination experiments of cisplatin and ^188^Re at specific concentration ratios are shown in Figure 3A. The logarithms of the survival were fitted by a linear function (L-Fit) in both cases. Compared to the curves for cisplatin and ^188^Re alone, the SF values for the combined treatment are significantly smaller. Approximately 2.4 Gy are required to achieve an SF value of 0.37, whereas only about 1.4 Gy is required for the combination with cisplatin. Isobolograms for the interaction of both drugs for a surviving cell fraction of 0.37 or 0.50 are shown in Figure 3B,C.

The corresponding survival for the combination of both drugs (red boxes in Figure 3B,C) is slightly above the 95% confidence interval of the cytotoxicity of irradiation and cisplatin alone, indicating that there was a sub-additive effect when cisplatin and ^188^Re were combined. Because we assumed linear dose–response curves for both cisplatin and ^188^Re, there is no difference between Mode I and II in the calculated lines of additivity in this isobolographic analysis (see Section 4).

#### 2.2.3. Combination of Cisplatin and External X-rays

**Figure 4:**
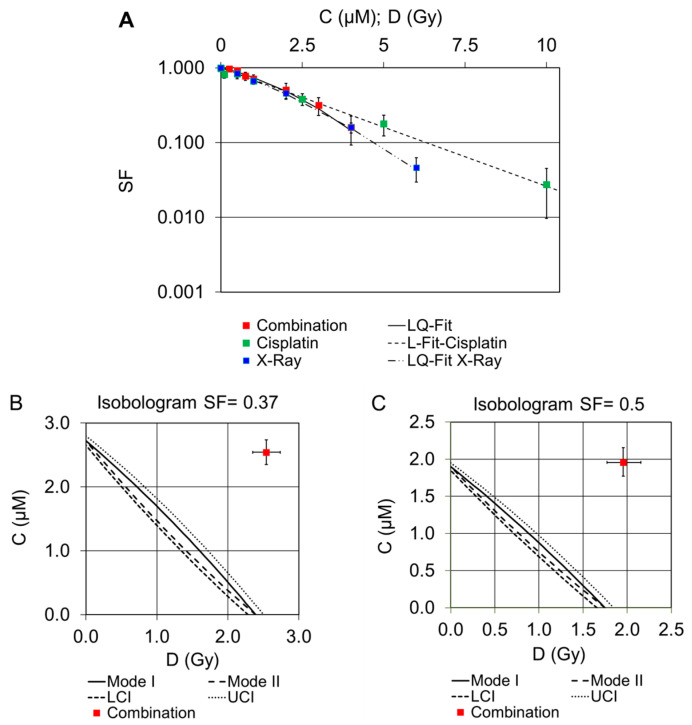
Survival curves of single and combined treatment experiments with cisplatin and X-rays are shown. Measurements are expressed as mean ± SD (**A**). Isobolograms show survival fractions of 0.37 (**B**) and 0.50 (**C**). The solid line represents the line of additivity; the dashed lines are the calculated errors with respect to the lower (LCI) and upper (UCI) 95% confidence intervals.

Combination experiments of cisplatin and external X-ray at specific concentration ratios are shown in Figure 4A. The survival curves for X-ray and the combination were best described by the linear–quadratic model. There are very similar proportions of surviving cells induced by X-rays and cisplatin alone, as well as for their combination at the respective dose points. Isobolograms for the interaction of both drugs are shown in Figure 4B,C. In the isobolograms, the envelope of additivity, i.e., the difference between Mode I and Mode II calculations, is clearly visible.

According to the results shown in Figure 4A, the combination of cisplatin and external X-rays did not lead to an additive effect (Figure 4B,C). In contrast, a clear protective effect was observed by interpreting the isobolograms (red boxes).

## 3. Discussion

In our study, we investigated the combined treatment using cisplatin and the radionuclides ^233^Ra, ^188^Re, as well as external X-rays. Head and neck tumor cells (FaDu) served as biological models. To interpret the results as supra-additive, additive, or protective, the concept of isobolographic analyses was used.

Looking at the results for the single treatment with ^223^Ra, the survival curve leads to the assumption that the alpha-emitter is more effective in cell eradication compared to ^188^Re and X-rays at similar doses. Ten times lower D_37_ and D_50_ values were observed for ^223^Ra compared to ^188^Re and X-rays. This is primarily due to the different mechanisms of DNA damage induction. While ^188^Re and X-rays induce strand breaks mainly via indirect effects and the generation of free radical species, ^223^Ra is capable of inducing direct DNA double-strand breaks (DSB) by emitting high-energy particles. Similar results were found for the alpha-emitter ^223^Ra compared to ^188^Re in PCCL3 cells [31]. Recently, our group published a study that investigated the effects of cisplatin in combination with radionuclides using plasmid pUC19 as a biophysical model. No significant increase in the number of DNA strand breaks has been found [32].

A study by Dewey et al. summarized that a significant amount of apoptosis recruits tumor cells into the apoptotic-susceptible fraction between daily external radiation doses [30]. The authors postulated that fractionated radiation therapy increases cell killing by apoptosis more than large single doses. This may be one explanation for the radioprotective effect of cisplatin in combination with a single dose of X-rays in our experiments.

In conclusion, our results of the combinatorial treatments showed a supra-additive effect only for the combination of cisplatin and ^223^Ra, which again could be caused by the mechanism of action of cisplatin and the already higher effect of ^223^Ra. In more detail, cisplatin molecules form adducts with nucleophilic sites of DNA, which can block DNA replication, transcription and damage repair [29]. Damage could also result from the emission of Auger electrons and photoelectrons generated by radiation in high-Z atoms such as platinum [22]. Thus, the enhancement of cell death in combined treatments is most likely provoked by the enhancement of irreparable damage to DNA, leading to an increase in initial lesions. Additionally, the high-LET emitter ^223^Ra may enhance cell death more efficiently than low-LET emitters such as ^188^Re caused by impaired repair of the DNA damage [28].

Geldof et al. demonstrated supra-additive treatment effects in prostate cancer cells by combining ^186^Re-HEDP and cisplatin [20]. The combined treatment of HepG2 tumor cells with ^131^I-NaI as a radiotherapeutic agent and cisplatin also resulted in improved cell death in a supra-additive manner [19], suggesting successful radiosensitizing effects when cisplatin is combined with low-LET emitters.

Recently, a preclinical and a clinical study evaluated combined chemo- and radionuclide therapy approaches. Timin et al. implemented these concepts using radionuclide carriers (^177^Lu-labeled core-shell particles) and cisplatin to treat metastatic lung cancer in animals [21]. This combination increased the therapeutic efficacy of tumor treatment compared to monotherapy. A clinical trial evaluated the efficacy of ^90^Y-transarterial radioembolization with cisplatin for the first-line treatment of locally advanced intrahepatic cholangiocarcinoma (iCCA). The authors found this approach to be an effective treatment for iCCA with a high rate of downstaging to tumor resection [18]. 

Many studies have investigated the combination of external beam radiation and a chemotherapeutic agent. Gorodetsky et al. showed both radiosensitizing and radioprotective effects depending on the chosen treatment sequence of the noxes X-ray and cisplatin [14]. Akudugu and Slabbert investigated the modulation of radiosensitivity by cisplatin in V79 Chinese hamster lung fibroblasts [33]. Their results show that the mode of interaction between cisplatin and gamma irradiation depends on the concentration and exposure time of cisplatin, as well as the timing of irradiation after cisplatin administration. Increased radiosensitivity was found when cisplatin was present in the cells for 8–12 h and 20–24 h. This experimental setting is in contrast to our study, which used a drug incubation interval of 4 h.

Additionally, the importance of different LET values on radiosensitizing effects with cisplatin has been investigated. Shiba et al. found that low-LET carbon–ion irradiation in combination with cisplatin produced higher cytotoxic effects than high-LET carbon–ion irradiation in cervical cancer cells [34]. On the other hand, carbon ion irradiation combined with cisplatin showed superior potential to kill breast cancer cells with irreparable DNA damage [35]. Benzina et al. combined cisplatin as well as oxaliplatin in two different studies with high-LET irradiation by p (65) + Be neutrons (dose rate 0.2 Gy/min) using glioblastoma cells. Their approaches enhanced the cytotoxicity in a more than additive way or caused a marked reduction in tumor growth in nude mice xenografts, respectively [28,36]. In addition, high-LET CIERT was more effective than photon irradiation in preventing the proliferation of HNSCC cell lines [37].

Overall, several studies have shown the improved efficacy of cancer therapy in vitro and in vivo, even when radionuclides are used in combination with cisplatin. Similarly, our in vitro study showed, at least for ^223^Ra in combination with cisplatin, improved tumor cell eradication, which can be interpreted as a supra-additive effect. We are aware that our results were obtained using the same combination protocol for all experiments. By changing the first and second cytotoxin, as described by some authors [8,14,33], such changes in the cytotoxin sequence could lead to results different from ours. Furthermore, in our experiments, cisplatin and radiotherapy treatments were administered only once, whereas realistic therapies for both cytostatic drugs and external irradiation are likely to be administered in cycles. Thus, it can be expected that the clinical effects of fractionated radiotherapy and chemotherapy applied in cycles may lead to different effects on tumor survival. Overall, experiments using in vitro cell models are only partly applicable to living organisms. Further studies should focus on potential clinical applications in nuclear medicine.

From a biological point of view, other endpoints, such as apoptosis or cell cycle analysis, might be helpful to gain more insights into the cellular response to combined chemo-radiotherapy. A better understanding of the molecular response of cells could lead to research based on treatments that combine pharmacological interventions with ionizing radiation to more specifically target tumor tissue, namely, multiple DNA repair pathways, cell cycle checkpoints, or modulation of signal transduction pathways [38].

For our study, it can be assumed that the radiation qualities other than ^223^Ra, namely ^188^Re and X-rays, could have similar effects when different experimental schemes are applied. This is something to be tested in more detail to complement this study, which has demonstrated that the applied statistical methods and settings used are well-suited to detect different drug interactions in this particular area of interest.

## 4. Materials and Methods

### 4.1. Radionuclides and X-ray Irradiation

The β-emitter ^188^Re-perrhenate (^188^ReO_4_^−^) was obtained by elution of a 40-GBq alumina-based ^188^W/^188^Re generator (Isotope Technologies Garching GmbH, Garching Germany). Physical properties of ^188^Re are half-life T_1/2_ = 17 h and maximum β-energy = 2.1 MeV.

The α-particle emitter ^223^Ra-radium dichloride (^223^RaCl_2_, Xofigo) was provided by Bayer Vital GmbH (Leverkusen, Germany) with an activity concentration of 1000 kBq/mL. ^223^Ra (half-life 11.4 days) decays through a cascade of short-lived α- and β-particle emitters. Each decay of ^223^Ra produces four α-particles, resulting in the emission of approximately 28 MeV of energy, with 95% of the energy from the α-emissions.

Each of the radioactive samples was measured with an Isomed 2010 (Nuvia Instruments, Dresden, Germany) dose calibrator.

For the external irradiations at the OncoRay site (National Center for Radiation Research in Oncology, Medical Faculty Dresden, Germany), an X-ray tube (Y.TU 320, Yxlon International, Hamburg, Germany) with 200 kV X-rays (20 mA, dose rate ≈ 1.24 Gy/min, filtered with 0.5 mm Cu) was used.

### 4.2. Cell Culture

FaDu cells are epithelial, squamous cell carcinoma cells of the pharynx. They were established in 1968 from a biopsy of an undifferentiated human squamous cell carcinoma growing as a monolayer (HTB-43^TM^, American Type Culture Collection (ATCC^®)^, Manassas, VA, USA) [39]. Our experiments were performed with the sub-cell line FaDu_DD_, kindly provided by the Department of Radiotherapy and Radiation Oncology, Medical Faculty, Technische Universität Dresden. This cell line has been used in cancer research, particularly in radiobiological experiments, since the 1980s. [40]. The cells were maintained in Dulbecco’s minimum essential medium (DMEM, Fisher Scientific, Wesel, Germany) containing 2% (*v*/*v*) HEPES buffer, 1% (*v*/*v*) of non-essential amino acids, 1% (*v*/*v*) of sodium pyruvate, and 10% (*v*/*v*) of fetal calf serum. All chemicals added to the cell culture medium were purchased from Sigma Aldrich (Sigma Aldrich, Taufkirchen, Germany). Exponentially growing cells were split twice weekly using trypsin (Sigma Aldrich) and cultured in a humidified incubator at 37 °C and 5% CO_2_. To prevent cell dedifferentiation, the experiments were performed at identical passage numbers. Cells were routinely screened for mycoplasma infection.

### 4.3. Cisplatin Incubation, Irradiation Procedure, and Colony Formation Assay

To study the cytotoxicity of cisplatin (Merck KGaA, Darmstadt, Germany), X-rays, and the radionuclides ^188^Re or ^223^Ra as single agents, 0.5 × 10^6^ FaDu cells were plated in each well of 6-well multititer plates (MTP) one day before the start of the experiment. To investigate the cytotoxic effects of cisplatin (0.01; 0.1; 0.5; 1.0; 2.5; 5.0; 10; and 20 µM), FaDu cells were treated for 4 h. The ^188^Re-, ^223^Ra-radioactive solutions, 0.69–16.5 MBq/mL, and 0.008–0.127 MBq/mL, respectively, were added to the cells to achieve doses of 0.25–6.0 Gy. In the case of X-ray irradiation, the cells were exposed to 0.5–6.0 Gy. Untreated control samples were included in each experiment.

After calculating the respective D_50_ and C_50_ values for each of the radionuclides and for cisplatin, the iso-effective doses or concentrations were chosen to establish the relationship between them. For the combined treatment experiments, we decided to use a relation of 1:1 for radionuclide doses and cisplatin concentrations (0.25; 0.5; 1.0; 2.0; 3.0; 4.0; 6.0 Gy or cisplatin in µM, factor 1.0). An exception was necessary for ^223^Ra due to the D_50_ value of 0.163 Gy. Therefore, the ratio of 0.125 Gy ^223^Ra to 1 µM cisplatin was used, resulting in a factor of 0.125. After a 2 h preincubation period with cisplatin alone, the ^188^Re- and ^223^Ra-radioactive solutions were added to the cells for a further 2 h incubation period to achieve dosages of 0.25–6.0 Gy or 0.25–6.0 µM, respectively. Similarly, for X-ray, cells were preincubated with cisplatin for 2 h and then exposed to 0.5–6.0 Gy (23.5–282 s). To ensure the same 4 h incubation time of cisplatin after X-ray irradiation, the incubation of the cells was continued for the remaining time window.

To determine the cytotoxic effects of the chemo-radio-therapeutic approach, the clonogenic cell survival was analyzed. Colony formation assays were performed as previously described [41]. Following irradiation, the radioactive supernatant was discarded, and the cells were washed with phosphate-buffered saline (PBS, 37 °C) and detached by trypsin. An aliquot of the cell suspension was seeded at a low density for colony formation at respective cell numbers adjusted to the doses or concentrations (200–50,000 cells) into T25 cell culture flasks (Greiner Bio-one, Frickenhausen, Germany) for an incubation period of 10 days. To stop colony formation, the cells were fixed in 80% (*v*/*v*) ethanol and stained with crystal violet solution. All chemicals were obtained by Merck KGaA.

Finally, cell colonies (>50 cells) were counted manually under a light microscope. The plating efficiency (PE) was calculated for treated and untreated cells based on the number of seeded cells. The surviving fraction (SF) was calculated as the relative plating efficiency of treated vs. untreated samples [42].

### 4.4. Isobologram Analysis

The possible interactions between radiation and chemotherapy were defined by Steel and Peckham [43]. For the isoeffective plots, the calculation of the theoretical lines of additivity was performed in two ways: Mode I and Mode II [26,43,44].

In short, Mode I assumes an independent action of the agents; the expected survival is the product of the individual survival for a given combination of concentration (cisplatin) and dose (irradiation). In Mode II, there may be an interaction between the agents, so an isoequivalent approach is performed. The first agent (cisplatin) causes damage, leading to surviving fraction (SF), and the radiation dose that would have had the same effect in an independent treatment is sought. It is then necessary to determine the additional dose that would have been required to achieve the desired level of survival. In the isobologram, the Mode I and II additivity curves are different and form an additivity envelope when the log survival curve is non-linear for at least one agent. In summary, this envelope refers to additive effects, but combined treatment could lead to supra-additive or protective effects.

### 4.5. Dosimetry

The dose calculation for radioactivity (^188^Re, ^223^Ra) was performed with Geant4 simulations for a 10 µm cell monolayer at the bottom of the well (9.6 cm^2^) in 2 mL cell culture medium. The dose from a source volume to a target volume is calculated as the product of the time-integrated activity in the source volume and a source-target-specific S value [31]. According to this model, only the extracellular irradiation of the medium was considered [45]. Thus, the applied X-ray radiation (external irradiation) is comparable to the dose of radioactivity generated.

For an effective dose of 1 Gy after 2 h of irradiation, the following activity concentrations were calculated: 5.5 MBq/2 mL ^188^Re, 0.127 MBq/2 mL ^223^Ra. The variation of the effective dose was achieved by increasing or decreasing the volume activity at a constant irradiation time of 2 h.

### 4.6. Statistical Analysis

All experiments were performed in three to four independent experiments. Triplicate samples were prepared for each dose point. The mean values of cell survival, including the calculated standard deviations, are presented against the respective cisplatin concentration or radiation dose. All data were statistically evaluated using the SPSS Statistics 24.0 software (IBM Corporation, Armonk, NY, USA). Curve fitting was performed by linear regression analysis.

In addition to standard statistics, isobolographic analyses were performed to better understand the drug–drug interaction of irradiation and cisplatin. For each treatment condition, the functional relationship between concentration or dose and the measured cell survival was analyzed according to the linear or linear–quadratic model. This curve fitting of log survival as dependent and either concentration or dose (and dose square) as independent variables was performed with the Linear Regression Tool of SPSS. The obtained regression coefficients for each treatment condition allowed the calculation of theoretical values of concentration and dose for a considered level of survival. 

The generation of isobolograms was performed in Microsoft EXCEL 2010. The envelope of additivity was calculated according to Section 4.4. from the experiments with single noxes of radiation and cisplatin. The combined treatment, in our case with a fixed ratio of concentration and dose, delivers a single point in the isobologram. Its position in relation to the additivity envelope was visually interpreted. To determine the uncertainties in terms of confidence levels, we repeated the isobolographic calculation using the upper and lower confidence intervals of the predicted values from the regression analyses. Therefore, the presented isobolograms show the effect of the combination experiments to interpret the overall cell survival as a function of these different drugs.

## 5. Conclusions

It has been shown that a general supra-additive effect is not to be expected after the combination of cisplatin and different radiation qualities. Nevertheless, the combination of ^223^Ra as a high-LET emitting radionuclide with the cytostatic drug cisplatin resulted in a supra-additive effect that can now be further evaluated. With the development of a well-functioning scheme for therapy at the cellular level, patients may also benefit from more knowledge about these combination approaches, and higher tumor-destroying effects may be achieved with lower radiation or drug doses.

## Figures and Tables

**Table 1:** Calculated C_37_ and C_50_ as well as D_37_ and D_50_ values for combined treatments of FaDu cells—mean value (95% confidence interval in brackets).

Treatment Conditions	C_50_ (µM)/D_50_ (Gy)	C_37_ (µM)/D_37_ (Gy)
Cisplatin (µM)	1.90 (1.85–1.95)	2.72 (2.65–2.80)
^223^Ra (Gy)	0.163 (0.156–0.169)	0.232 (0.223–0.242)
^188^Re (Gy)	1.69 (1.61–1.78)	2.42 (2.31–2.55)
X-ray (Gy)	1.75 (1.66–1.85)	2.39 (2.28–2.50)

## Data Availability

Data can be requested by contacting the corresponding author.
